# Viral Respiratory Tract Infections in Adult Patients Attending Outpatient and Emergency Departments, Taiwan, 2012–2013: A PCR/Electrospray Ionization Mass Spectrometry Study

**DOI:** 10.1097/MD.0000000000001545

**Published:** 2015-09-25

**Authors:** Hsin-I Shih, Hsuan-Chen Wang, Ih-Jen Su, Hsiang-Chin Hsu, Jen-Ren Wang, Hsiao Fang Sunny Sun, Chien-Hsuan Chou, Wen-Chien Ko, Ming-I Hsieh, Chi-Jung Wu

**Affiliations:** From the Departments of Emergency Medicine (H-IS, H-CH); Public Health (H-IS); Internal Medicine, National Cheng Kung University Hospital, College of Medicine, National Cheng Kung University (C-HC, W-CK, C-JW); National Institute of Infectious Diseases and Vaccinology, National Health Research Institutes (H-CW, I-JS, J-RW, M-IH, C-JW); Department of Medical Laboratory Science and Biotechnology (J-RW); and Institute of Molecular Medicine, College of Medicine, National Cheng Kung University, Tainan, Taiwan (HSS).

## Abstract

Viral etiologies of respiratory tract infections (RTIs) have been less studied in adult than in pediatric populations. Furthermore, the ability of PCR/electrospray ionization mass spectrometry (PCR/ESI-MS) to detect enteroviruses and rhinoviruses in respiratory samples has not been well evaluated. We sought to use PCR/ESI-MS to comprehensively investigate the viral epidemiology of adult RTIs, including testing for rhinoviruses and enteroviruses.

Nasopharyngeal or throat swabs from 267 adults with acute RTIs (212 upper RTIs and 55 lower RTIs) who visited a local clinic or the outpatient or emergency departments of a medical center in Taiwan between October 2012 and June 2013 were tested for respiratory viruses by both virus isolation and PCR/ESI-MS. Throat swabs from 15 patients with bacterial infections and 27 individuals without active infections were included as control samples.

Respiratory viruses were found in 23.6%, 47.2%, and 47.9% of the 267 cases by virus isolation, PCR/ESI-MS, and both methods, respectively. When both methods were used, the influenza A virus (24.3%) and rhinoviruses (9.4%) were the most frequently identified viruses, whereas human coronaviruses, human metapneumovirus (hMPV), enteroviruses, adenoviruses, respiratory syncytial virus, and parainfluenza viruses were identified in small proportions of cases (<5% of cases for each type of virus). Coinfection was observed in 4.1% of cases. In the control group, only 1 (2.4%) sample tested positive for a respiratory virus by PCR/ESI-MS. Patients who were undergoing steroid treatment, had an active malignancy, or suffered from chronic obstructive pulmonary disease (COPD) were at risk for rhinovirus, hMPV, or parainfluenza infections, respectively. Overall, immunocompromised patients, patients with COPD, and patients receiving dialysis were at risk for noninfluenza respiratory virus infection. Rhinoviruses (12.7%), influenza A virus (10.9%), and parainfluenza viruses (7.3%) were the most common viruses involved in the 55 cases of lower RTIs. The factors of parainfluenza infection, old age, and immunosuppression were independently associated with lower RTIs.

In conclusion, PCR/ESI-MS improved the diagnostic yield for viral RTIs. Non-influenza respiratory virus infections were associated with patients with comorbidities and with lower RTIs. Additional studies that delineate the clinical need for including non-influenza respiratory viruses in the diagnostic work-up in these populations are warranted.

## INTRODUCTION

Viral respiratory tract infections (RTIs) in humans occur throughout the year and represent a major cause of clinical visits worldwide. In the past, the viral causes of RTIs were largely unknown, primarily due to the insensitivity of culture-based methods for the detection of viruses or to the narrow spectrum of viral detection using singleplex nucleic acid tests (NATs). Recently, the development of multiplex respiratory NATs has allowed for the simultaneous, rapid, and sensitive detection of multiple viruses, which facilitates comprehensive studies regarding the epidemiology of viral RTIs. Currently, the viral epidemiology of RTIs has been studied more extensively among pediatric populations compared with adult populations throughout the world.^1^ Similarly, most studies describing the viral etiology of respiratory illness in Taiwan, a subtropical country in Eastern Asia, were limited to pediatric populations.^2–4^ Thus, studies among adult patients are lacking, particularly regarding infections due to fastidious or newly identified viruses, such as human metapneumovirus (hMPV) and human coronavirus (hCoV). Overlapping clinical presentations shared by different respiratory viruses make differential diagnoses difficult to perform based solely on the clinical parameters.^5^ Moreover, effective antiviral agents are currently restricted to influenza virus infections. Hence, a better understanding of the epidemiology of adult viral RTIs would aid the future design of diagnostic strategies, infection control, and patient management.

Among the various multiplex NATs, multilocus polymerase chain reaction coupled with electrospray ionization mass spectrometry (PCR/ESI-MS) can simultaneously identify and subtype multiple respiratory viruses.^6–9^ Despite the diagnostic potential, the ability of PCR/ESI-MS to detect human enterovirus and rhinovirus in respiratory samples from patients with RTIs has not been well evaluated. Previous PCR/ESI-MS studies in patients with RTIs did not include these 2 viruses in the diagnostic panels.^6–9^ Here, we expanded upon these previous studies utilizing PCR/ESI-MS for respiratory virus detection. We aimed to comprehensively investigate the epidemiology of adult viral RTIs using PCR/ESI-MS and compare the diagnostic performance between PCR/ESI-MS and conventional culture methods for identifying multiple, clinically relevant, respiratory viruses, including enterovirus and rhinovirus.

## METHODS

### Patients and Specimens

To conduct a comprehensive epidemiologic study that included patients with and without comorbidity, we enrolled adults (of at least 18 yr of age) with acute RTIs within 7 days of onset who were treated at a local outpatient clinic of YC hospital or the outpatient or emergency departments of National Cheng-Kung University Hospital (NCKUH), a university-affiliated medical center in southern Taiwan, between October 2012 and June 2013. Acute RTI was defined as the simultaneous occurrence of at least 1 respiratory symptom or sign (new or worsening cough, sputum production, sore throat, nasal congestion, rhinorrhea, dyspnea, wheezing, or injected tonsils) and at least 1 of the following symptoms: fever, chills, and cough. Lower RTI (LRTI) was defined as the presence of acute RTI and a new infiltrate on chest radiograph. For patients experiencing more than 1 episode of RTI, the most recent episode was counted as separate only if the patient fully recovered from the previous episode and there was a least a 3-week interval between the onset of the 2 episodes. Clinical, laboratory, and radiological data and the contact history of each patient were retrieved. Comorbidities were assessed in all patients based on the Charlson comorbidity index (CCI).^10^ Steroid use was defined as the receipt of corticosteroid treatment (10 mg prednisolone or an equivalent daily dosage) for more than 2 weeks. An immunocompromised state was diagnosed if the patients met one of the following conditions: corticosteroid treatment, solid organ or hematopoietic stem cell recipient, or chemotherapy for an underlying malignancy during the past 6 months.

Nasopharyngeal or throat swabs were obtained from all patients and collected in transport medium, as previously described.^11^ for virus detection and identification by both virus isolation and PCR/ESI-MS. Clinical specimens were stored at 4°C and transported to the study sites within 24 hours of collection. Throat swabs from 42 cases without respiratory infections during the month prior to enrollment were included as control samples for PCR/ESI-MS analysis, including 15 patients with exclusively bacterial infections (documented cases of bacteremia or urinary tract infection) who were admitted to NCKUH and 27 individuals without active infections. These subjects without active infections included 10 patients with stable chronic diseases followed up in NCKUH clinics and 17 healthy individuals whose medical information was collected using a clinical questionnaire.

The study was approved by the Institutional Review Board (B-ER-101-031) of the study hospital, and all patients provided informed consent.

### Virus Isolation and Identification

Respiratory specimens were inoculated onto appropriate tissue cultures (Madin–Darby canine kidney, MRC-5, A549, and rhabdomyosarcoma) to isolate human influenza virus, parainfluenza virus, genus *Enterovirus*, cytomegalovirus (CMV), adenovirus, respiratory syncytial virus (RSV), herpes simplex viruses 1 and 2 (HSV-1 and -2), and varicella zoster virus (VZV). The isolation and identification of viruses were performed using a previously described method^11^ and enteroviruses were identified by a immunofluorescence assay using a Chemicon Pan EV mix that cross-reacts with rhinovirus (Light Diagnostics, Chemicon [Millipore], MA).^11,12^

### Virus Detection and Identification by PCR/ESI-MS

Total nucleic acids were extracted from 700 μL of swab samples using a nucleic acid autoextractor (MagNA Pure Compact Instrument, Mannheim, Germany), and the eluate was stored at −80°C until analysis. During the analyses, the extracted nucleic acids were added to both a PLEX-ID Respiratory Virus assay plate and a PLEX-ID Broad Viral I assay plate (PLEX-ID, Abbott Laboratories, Abbott Park, Illinois). The PLEX-ID Respiratory Virus assay detects human adenovirus, hCoV, hMPV, influenza A and B, parainfluenza types 1 to 3, and RSV,^6^ whereas the PLEX-ID Broad Viral I assay detects human adenovirus, enterovirus, rhinovirus, BK and JC polyomavirus, parvovirus B19, HSV-1 and -2, VZV, Epstein–Barr virus (EBV), CMV, and human herpesvirus (HHV)-8.^13,14^ In this study, respiratory viruses refer to adenovirus, hCoV, hMPV, influenza, parainfluenza, RSV, enterovirus, and rhinovirus. Nucleic acid amplification and analyses of PCR products were conducted using the PCR/ESI-MS platform (PLEX-ID, Abbott Laboratories) following the manufacturer's instructions, with test turnaround time from sample to result within 6 to 8 hours.^8,13^ The PCR/ESI-MS analyses included automated PCR desalting, ESI-MS signal acquisition, spectral analysis, and data reporting. Organism identification was based on the total mass and base compositions of the PCR amplicons compared with those in the molecular signature database established by the PLEX-ID manufacturer.^6,8,13,14^

Samples in which PCR/ESI-MS results disagreed with culture results at the species level were reexamined by a second molecular method. For enteroviruses, rhinovirus was differentiated from enterovirus using a conventional PCR sequencing analysis with the previously described primers (Rhinovirus s1 and as) and a BLAST search.^15^

## STATISTICS

All analyses were performed with the Statistical Package for the Social Sciences version 17.0 (SPSS Inc, Chicago, IL). Continuous variables were expressed as mean values ± standard deviations and were compared using the analysis of variance test. Categorical variables were compared using the Fisher exact test or *χ*^2^ test. All biologically plausible variables with a *P* value ≤0.10 in the univariate analysis were considered for inclusion in the logistic regression model for the multivariate analysis. A *P* value less than 0.05 was considered statistically significant, and all tests were 2-tailed.

## RESULTS

### Patients

During the 9-month study period, a total of 267 episodes of acute RTIs from 263 patients were recorded, including 96 episodes at a local clinic and 171 episodes at NCKUH (19 outpatient and 152 in the emergency departments). For convenience, each episode was counted as 1 case. Overall, 123 (46.1%) cases were male patients, and 152 (56.9%), 60 (22.5%), and 55 (20.6%) patients were 18 to 39, 40 to 59, and ≥60 years of age, respectively. Two-hundred and twelve (79.4%) patients presented with upper RTIs (URTIs), and 55 (20.6%) cases presented with LRTIs. Compared with patients attending the local clinic, patients attending the medical care center were older and had more comorbidities (Table 1). The detailed demographic data of the 267 RTI cases and 42 control cases are presented in Table 1.

**TABLE 1:**
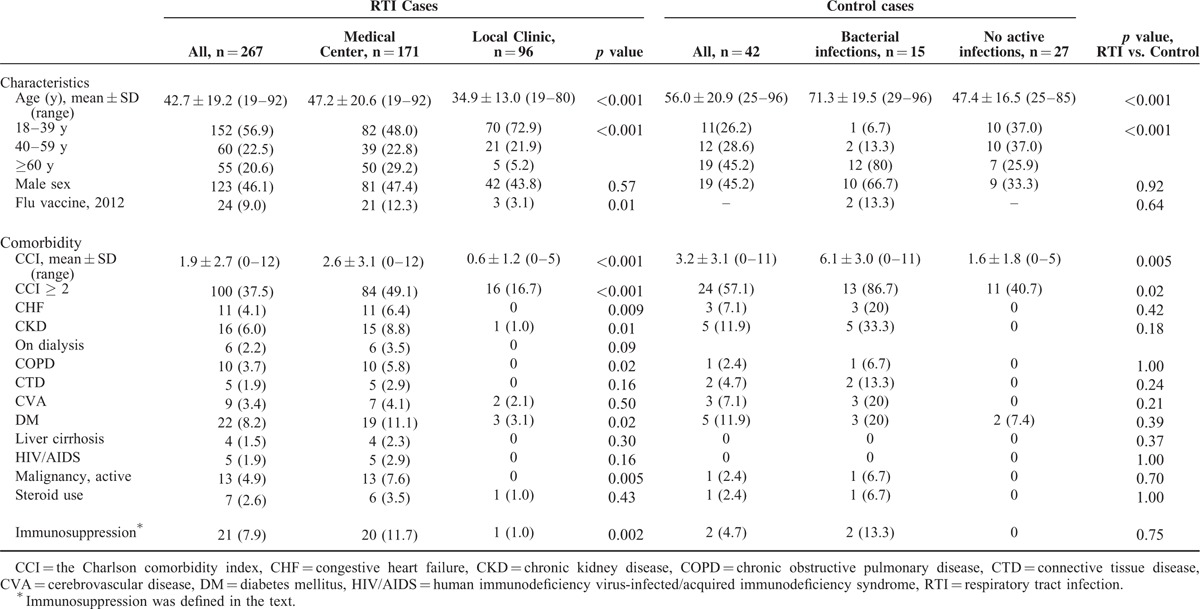
Demographic Characteristics of Patients With Acute Respiratory Tract Infections, Patients With Exclusively Bacterial Infections, and Individuals Without Active Infections Examined Between October 2012 and June 2013

### Virus Detection by Culture

All 267 respiratory samples from each RTI case were examined for viruses by both virus isolation and PCR/ESI-MS, and the results are presented in Table 2. For virus isolation, respiratory viruses were detected in 63 (23.6%) cases, including influenza A (48 cases, 18.0%), enterovirus (13, 4.9%), and parainfluenza virus (2, 0.7%), and no coinfection was detected. Virus isolation identified additional parainfluenza type 3 and enterovirus infections that were not found by PCR/ESI-MS in 2 samples.

**TABLE 2:**
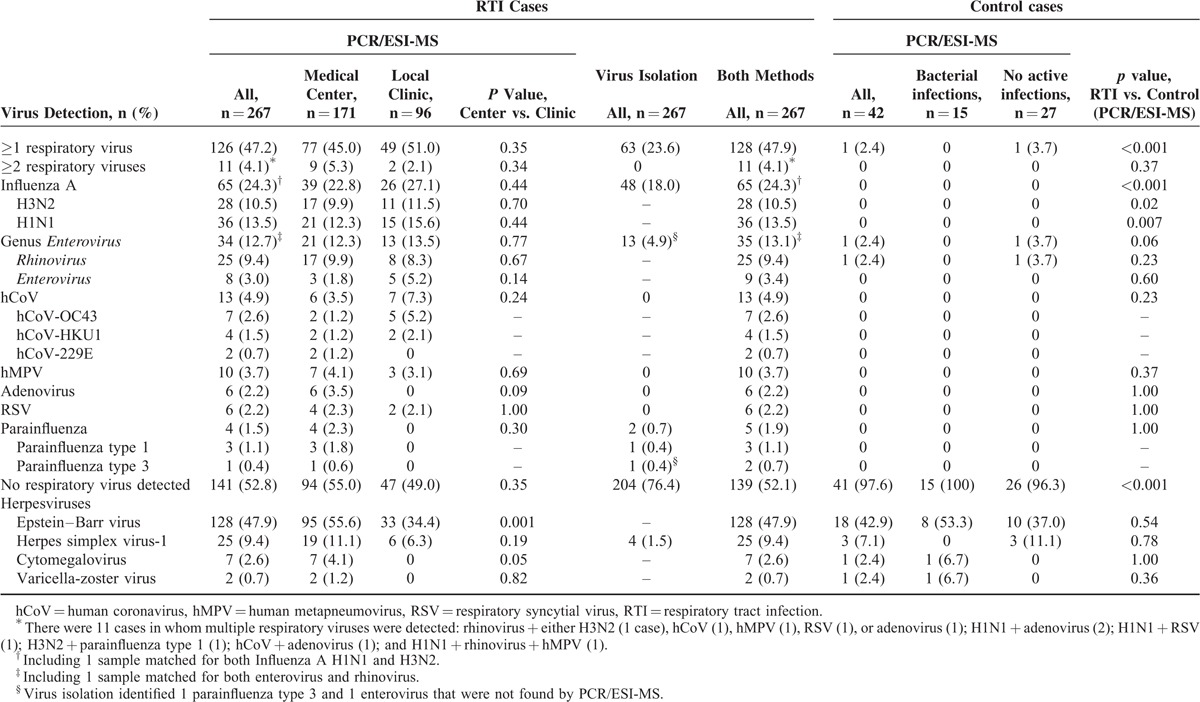
Frequencies of Virus Detection by PCR/ESI-MS and Virus Isolation Among Patients With Acute Respiratory Tract Infections, Patients With Exclusively Bacterial Infections, and Individuals Without Active Infections

### Virus Detection by PCR-ESI/MS

By PCR/ESI-MS, respiratory viruses were detected in 126 cases (47.2%). Influenza A (65 cases, 24.3%) was the most frequently identified virus, among which 36 (13.5%) cases were subtyped as pandemic H1N1/09 virus, 28 (10.5%) cases as seasonal H3N2 virus, and 1 case as influenza A matching both pandemic H1N1and seasonal H3N2. Genus *Enterovirus* (34, 12.7%) was the second-most frequently detected virus, including rhinovirus (25, 9.4%), enterovirus (8, 3.0%), and 1 culture-negative case matching for both rhinovirus and enterovirus. hCoV (13, 4.9%), hMPV (10, 3.7%), adenovirus (6, 2.2%), RSV (6, 2.2%), and parainfluenza (4, 1.5%) were detected in small proportions of cases. Simultaneous detection of more than 1 respiratory virus was observed in 11 (4.1%) patients, and rhinovirus (5 cases) was most likely to be codetected with another respiratory virus (Table 2). Of note, 4 cultivated viruses identified as enterovirus because of reactivity with the Chemicon Pan EV mix were characterized as rhinovirus by PCR/ESI-MS. Further PCR-sequencing analysis of the 4 clinical specimens confirmed the existence of rhinoviruses but not enteroviruses. PCR/ESI-MS identified additional respiratory viruses in 65 culture-negative samples, mostly rhinovirus (21 samples), and a second respiratory virus in 3 culture-positive influenza A samples. Overall, the positive detection rates for any respiratory virus by culture, PCR/ESI-MS, and both methods were 23.6%, 47.2%, and 47.9% (128/267), respectively. Of 61 specimens positive by both methods, PCR/ESI-MS and culture methods reached levels of agreement of 100% at the species level for influenza and parainfluenza and 100% at the genus level for the genus *Enterovirus*. In the control group, only 1 (2.4%) healthy individual tested positive for a respiratory virus (rhinovirus) by PCR/ESI-MS.

With respect to herpesviruses, PCR/ESI-MS identified EBV, HSV-1, CMV, and VZV in 128 (47.9%), 25 (9.4%), 7 (2.6%), and 2 (0.7%) samples from RTI cases, with similar detection rates observed in the control group. There was no detection of polyomavirus, parvovirus B19, HSV-2, or HHV-8 virus in samples from cases with RTIs or the control group.

### Distribution of Respiratory Viruses

**FIGURE 1:**
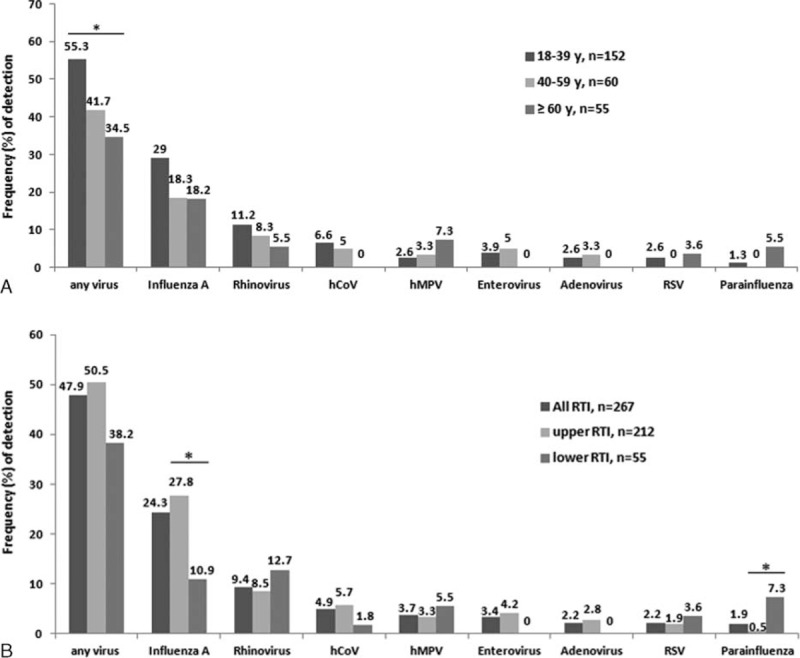
Frequency of respiratory virus detection according to (A) age groups and (B) clinical diseases (upper and lower respiratory tract infection [RTI]) by a combination of PCR/ESI-MS and virus isolation. hCoV = human coronavirus, hMPV = human metapneumovirus, RSV = respiratory syncytial virus. ^∗^*P* < 0.05.

Cases that tested positive for any respiratory virus either by culture or by PCR/ESI-MS were analyzed. The positive detection rates declined with age: 55.3%, 41.7%, and 34.5% in the 18–39, 40–59, and ≥60-year-old groups, respectively (*P* = 0.02) (Figure 1A). A higher positivity rate was observed in patients with URTIs than that in patients with LRTIs (50.5% vs. 38.2%, *P* = 0.10) (Table 3 and Figure 1B). There were similar distributions of respiratory viruses in cases from the local clinical and the medical center (Table 2), and between patients from the 3 age groups (Figure 1A). Of 128 cases with identifiable respiratory viruses, non-influenza virus infection was more common in patients with LRTIs than those with URTIs (81.0% [17/21] vs. 48.6% [52/107], *P* = 0.007). Rhinovirus (12.7%), influenza A (10.9%), and parainfluenza (7.3%) were the 3 leading respiratory viruses involved in 55 cases of LRTIs, and parainfluenza was more frequently observed in the LRTI group than in the URTI group (Table 3 and Figure 1B). There was no seasonal variation in any individual respiratory virus over the 9-month period.

**TABLE 3:**
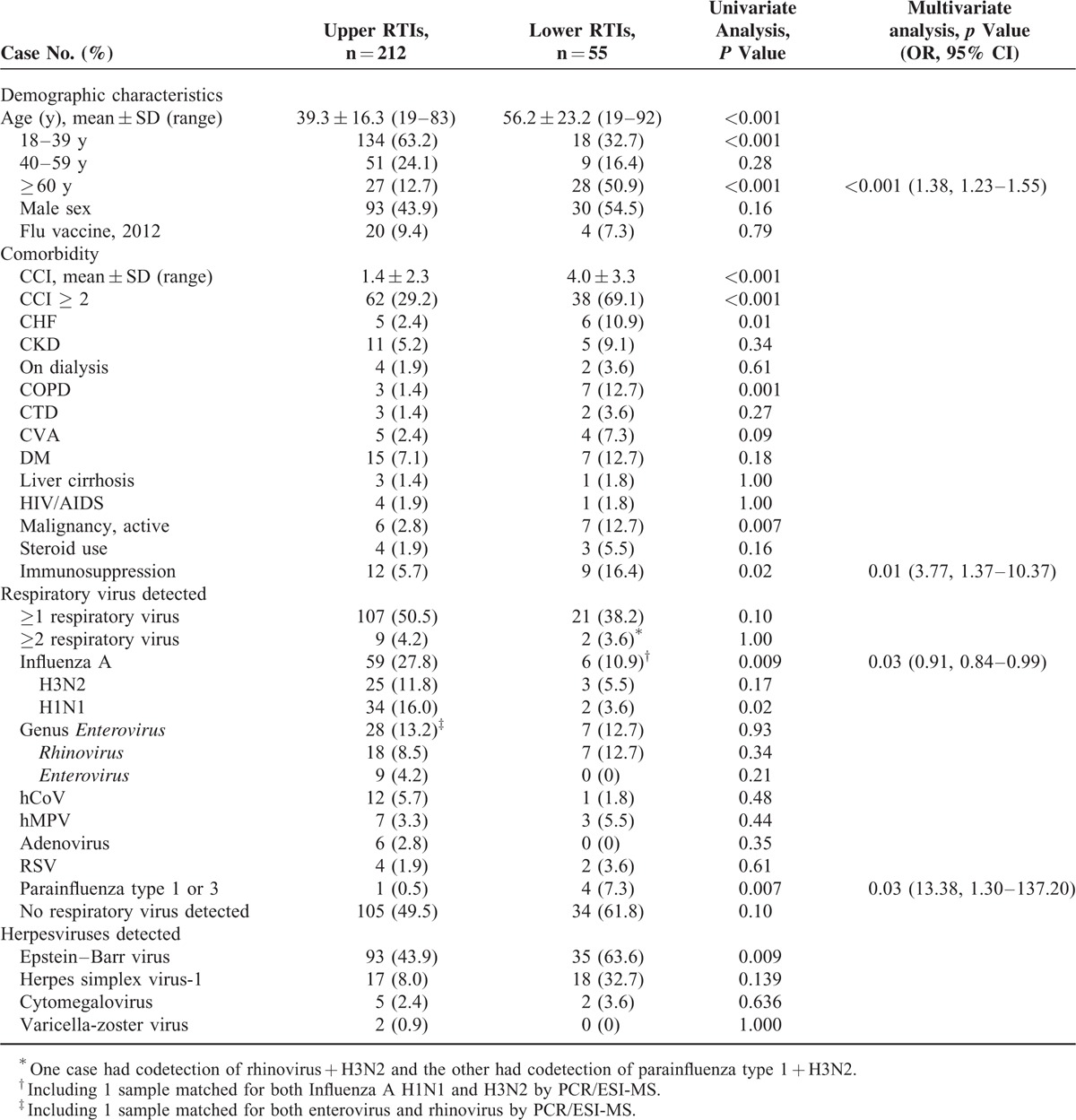
Demographic Characteristics and Respiratory Virus Distribution Determined by a Combination of PCR/ESI-MS and Virus Isolation in Patients With Upper and Lower Respiratory Tract Infections (RTIs) and Factors Associated With Lower RTIs

### Factors Associated With Respiratory Virus Detection and LRTIs

Of 128 patients with identifiable respiratory viruses, univariate analysis revealed that patients with 1 of the following conditions were more likely to have non-influenza respiratory virus infections: immunocompromised state, chronic obstructive pulmonary disease (COPD), and chronic renal failure receiving dialysis (OR 5.4, 95% CI 1.2–25.5, *P* = 0.02). Multivariate analysis demonstrated that steroid use was an independent risk factor for rhinovirus infection (OR 15.3, 95% CI 1.5–154.7, *P* = 0.02), active malignancy was an independent risk factor for hMPV infection (OR 29.3, 95% CI 2.4–358.1, *P* = 0.008), and COPD was an independent risk factor for parainfluenza infection (OR 229.2, 95% CI 10.5–5020.8, *P* = 0.001).

While comparing the URTI and LRTI groups, factors found to be associated with LRTI by univariate analysis included old age (≥60 years), a high comorbidity index, congestive heart failure, COPD, malignancy, immunocompromised state, and detection of parainfluenza or EBV, whereas detection of influenza A was less frequently associated with LRTI. Codetection of respiratory virus was not associated with the development of LRTI. By multivariate analysis, only old age, immunocompromised state, and detection of parainfluenza remained 3 independent factors associated with LRTI (Table 3).

### Clinical Data

Among the 117 episodes of single respiratory virus infections, arthralgia was more frequently observed in influenza A infections than in non-influenza infections (66.1% [39/59] vs. 46.6% [27/58], *P* = 0.033); for these 2 types of infections, the other examined symptoms, including sore throat, rhinorrhea, cough, purulent sputum, wheezing, dyspnea, and headache, were detected at similar frequencies.

Of 55 cases of LRTIs, coinfection with bacterial pathogens by sputum culture or blood culture was found in 3 (8.8%) of 34 patients who tested positive for respiratory viruses and in 2 (9.5%) of 21 patients who tested negative for respiratory viruses. Four of 6 cases of influenza A LRTI had received oseltamivir. Two patients died of pneumonia and the worsening of an underlying malignancy; 1 of these patients tested positive for hMPV, and the other patient tested negative for a respiratory virus. Four (16.7%) out of 24 patients vaccinated for influenza vaccine in 2012–2013 (A/California/7/2009 (H1N1)-like virus A/Victoria/361/2011 (H3N2)-like virus B/Wisconsin/1/2010-like virus) and 61 (25.1%) out of 243 unvaccinated patients had influenza A infections (*P* = 0.358).

## DISCUSSION

Our study of the viral epidemiology of adult acute RTI using PCR/ESI-MS technology has 3 major advantages. First, we expanded on previous studies utilizing PCR/ESI-MS for respiratory virus detection. The PLEX-ID Broad Viral I assay, which targets enterovirus, rhinovirus, herpesviruses, JC and BK polyomaviruses, and parvovirus B19, and the PLEX-ID Respiratory Virus assay tests were both adopted for the detection of multiple clinically relevant respiratory viruses. Second, 2 control groups (patients with exclusively bacterial infections and individuals without active infections) were enrolled to eliminate false-positive artifacts of NATs and estimate the prevalence of detectable asymptomatic carriers of respiratory viruses. Third, this study enrolled immunocompetent and immunocompromised patients visiting a local clinic or a medical center who presented with an URTI or LRTI, which reflects the true viral epidemiology of adult RTIs.

By supplementing the conventional culture method with PCR/ESI-MS, a 2-fold increase in the respiratory virus detection rate was achieved, from 23.6% by culture alone to 47.9% by a combination of both methods. Diagnostic gain was observed for both culturable viruses, especially rhinovirus, and fastidious viruses. Although we did not compare an alternative NAT due to sample volume limitations, it has been reported that PCR/ESI-MS has a high sensitivity (92.9–100%) and specificity (99–100%) for variable respiratory virus detection relative to immunologic and PCR-based methods as gold standard assays, with the exception of parainfluenza (sensitivity 63.4%).^6^ Coincidentally, we found that parainfluenza type 3 was 1 of only 2 viruses that were not detected by PCR/ESI-MS. The potential causes contributing to the lower detection rate for parainfluenza remain to be explored.

The positive detection rate (47.2%) for respiratory viruses by PCR/ESI-MS in the present study was similar to those of parallel adult surveillance programs using NATs (43.2–57%).^5,16–18^ but notably higher than an earlier study using the Ibis T5000 biosensor system (the prototype of PCR-ESI/MS) using the respiratory virus surveillance II kit (35.9%), likely because the kit was not designed for the detection of enterovirus and rhinovirus.^8^ Enterovirus and rhinovirus, both members of the *Enterovirus* genus, contributed to 13.1% of RTI cases in our study and 9.8–17.8% of adult cases in other studies.^5,16,17^ Considering their prevalence, enterovirus and rhinovirus should be included in the diagnostic panels of respiratory viruses if comprehensive viral detection is indicated. The codetection rate (4.1%) was within the range of 2.0–7.2% that has been reported elsewhere.^5,16,17^ and rhinovirus was the virus most frequently involved in coinfections, probably due to its high prevalence throughout the year.^18^

Influenza A and rhinovirus were the 2 most frequently detected respiratory viruses, whereas hCoV, hMPV, enterovirus, adenovirus, RSV, and parainfluenza were detected in small proportions of cases. This finding is similar to the viral epidemiology of adult RTIs observed by other study groups.^5,16,17^ The similar distributions of viruses between cases from a local clinic and a medical center and between patients of the 3 age groups suggest that individuals of all age groups are susceptible to multiple respiratory viruses that simultaneously circulate in the community. A lower positive detection rate was observed in the elderly population, probably because older adult patients shed lower titers of viruses.^19^ However, the roles of EBV, HSV-1, and CMV in adult RTIs remain incompletely elucidated; in particular, these viruses were detected in the control groups and the RTI groups at similar frequencies and can be asymptomatically shed from the oral mucosa or reactivated under physical stress.^20^ Moreover, the univariate association between EBV and LRTIs observed in this study may have been caused by the confounding factor of age, particularly given that old age was identified as an independent factor for EBV detection (data not shown). The lack of detection of BK and JC polyomavirus or parvovirus B19 implies that these viruses play a minor role in adult RTIs and that oropharyngeal cells are not involved in BK and JC polyomavirus persistence.^21^ Furthermore, the low positive detection rate for respiratory viruses in the control group suggests a low possibility of false-positive artifacts in PCR/ESI-MS or a lower rate of asymptomatic colonization of respiratory viruses.

In addition to the advantage of sensitive detection, PCR/ESI-MS possesses the capability of simultaneous subtype identification of respiratory viruses.^22^ In this study, influenza A viruses were subtyped as pandemic H1N1 influenza A and seasonal H3N2 influenza. In Europe, both viruses cocirculated in the community in the 2012–2013 influenza season.^23^ In the genus *Enterovirus*, acid-labile rhinovirus can be differentiated from enterovirus using an acid lability test.^24^ while PCR/ESI-MS can rapidly differentiate the 2 species in a single test, as demonstrated in our study. The 13 hCoVs were subtyped as hCoV-OC43, -229E, and -HKU1, which was further validated by conventional PCR-sequencing assays (data not shown). The newly identified HCoV-NL63 was not detected during the study period, and a low detection rate (<1%) was reported in China.^16^

Our understanding of the roles of non-influenza respiratory viruses in patients with comorbidities or LRTIs has been strengthened in our study. Patients who were undergoing steroid treatment, had an active malignancy, or suffered from COPD were at risk for rhinovirus, hMPV, or parainfluenza infections, respectively. Overall, immunocompromised patients, those with COPD, and patients receiving dialysis were at risk for non-influenza respiratory virus infection. Non-influenza virus infections were also more frequently involved in LRTIs than in URTIs. Among LRTIs, rhinovirus and parainfluenza were ranked as the first- and third-most common pathogens, respectively, and parainfluenza was an independent factor associated with LRTIs, a finding consistent with prior reports that both viruses are significant causes of LRTIs.^18,25–27^ On the other hand, despite an increasing role of non-influenza respiratory viruses, currently available antiviral agents and vaccines primarily target influenza infection. Although viral RTI is a self-limited illness, as observed in the majority of our patients with LRTIs who recovered from illness without the aid of antiviral agents, a definite etiological diagnosis can help to reduce the unwarranted use of anti-influenza agents or antimicrobials and/or unnecessary hospitalizations, and provide useful information for the control of RTIs. However, we observed that clinical differentiation of influenza infection from other respiratory virus infections is difficult due to overlapping symptoms, as described previously.^5^ Collectively, the association of non-influenza virus infection with patients with comorbidities or LRTIs reported here suggests that a complete respiratory viral panel would be appropriate in the diagnostic work-up for RTIs in these populations. The additional costs incurred by the use of a complete panel of PCR/ESI-MS-based assessments or other molecular tests would likely be offset by the accompanying reductions in unnecessary antimicrobial therapy and/or hospitalization.^18^

Our study has some limitations. First, parainfluenza type 4 and 3 newly identified respiratory viruses, human bocavirus, human polyomavirus KI and WU polyomavirus were not included in the panels.^28–31^ and their roles in adult RTIs in Taiwan are unclear. Second, although certain risk factors for specific virus infections, such as hMPV or parainfluenza infections, have been identified, these associations should be re-examined in additional large-scale clinical studies, and the clinical impact and underlying mechanisms of these associations should be explored. Similarly, more control cases may be needed to better estimate the prevalence of asymptomatic carriers of respiratory viruses. Third, only 3 seasons were covered, and the seasonality of viral respiratory infections could not be demonstrated.

In conclusion, compared with virus isolation, PCR/ESI-MS produced a greater diagnostic yield for viral RTIs, with a low possibility of false-positive artifacts. Non-influenza respiratory virus infection was significantly associated with patients with comorbidities and with LRTIs. Additional studies to delineate the clinical need for and economic benefits of including non-influenza respiratory viruses in the diagnostic work-up in these populations are warranted.
